# Effect of *phyllanthus niruri* on metabolic parameters of patients with kidney stone: a perspective for disease prevention

**DOI:** 10.1590/S1677-5538.IBJU.2017.0521

**Published:** 2018

**Authors:** Nidia D. Pucci, Giovanni S. Marchini, Eduardo Mazzucchi, Sabrina T. Reis, Miguel Srougi, Denise Evazian, William C. Nahas

**Affiliations:** 1 Divisão de Nutrição e Dietética, Instituto Central, Hospital das Clínicas, Universidade de São Paulo, Faculdade de Medicina. São Paulo, Brasil;; 2 Divisão de Urologia, Hospital das Clínicas, Universidade de São Paulo, Faculdade de Medicina, São Paulo, Brasil;; 3 Laboratório de Investigação Médica, Universidade de São Paulo, Faculdade de Medicina, São Paulo, Brasil

**Keywords:** Kidney Calculi, Disease, Urolithiasis

## Abstract

*Phyllanthus niruri (P.niruri) o*r stone breaker is a plant commonly used to reduce stone risk, however, clinical studies on this issue are lacking.

**Objective:**

To prospectively evaluate the effect of *P. niruri* on the urinary metabolic parameters of patients with urinary lithiasis.

**Materials and Methods:**

We studied 56 patients with kidney stones <10mm. Clinical, metabolic, and ultrasonography assessment was conducted before (baseline) the use of *P. niruri* infusion for 12-weeks *(P. niruri)* and after a 12-week (wash out) Statistical analysis included ANOVA for repeated measures and Tukey's/McNemar's test for categorical variables. Significance was set at 5%.

**Results:**

Mean age was 44±9.2 and BMI was 27.2±4.4kg/m2. Thirty-six patients (64%) were women. There were no significant changes in all periods for anthropometric and several serum measurements, including total blood count, creatinine, uric acid, sodium, potassium, calcium, urine volume and pH; a significant increase in urinary potassium from 50.5±20.4 to 56.2±21.8 mg/24-hour (p=0.017); magnesium/creatinine ratio 58±22.5 to 69.1±28.6mg/gCr24-hour (p=0.013) and potassium/creatinine ratio 39.3±15.1 to 51.3±34.7mg/gCr24-hour (p=0.008) from baseline to wash out. The kidney stones decreased from 3.2±2 to 2.0±2per patient (p<0.001). In hyperoxaluria patients, urinary oxalate reduced from 59.0±11.7 to 28.8±16.0mg/24-hour (p=0.0002), and in hyperuricosuria there was a decrease in urinary uric acid from 0.77±0.22 to 0.54±0.07mg/24-hour (p=0.0057).

**Conclusions:**

*P.niruri* intake is safe and does not cause significant adverse effects on serum metabolic parameters. It increases urinary excretion of magnesium and potassium caused a significant decrease in urinary oxalate and uric acid in patients with hyperoxaluria and hyperuricosuria. The consumption of *P.niruri c*ontributed to the elimination of urinary calculi.

## INTRODUCTION

Risk factors for urolithiasis include congenital, genetic, environmental, dietary and metabolic aspects; chronic diseases including obesity, hypertension and diabetes are also associated with urinary calculus formation (1). Overall, urinary calculi derive from a combination of some of the factors involved in its pathophysiology (1). The worldwide prevalence of urinary calculi is 8.8% and is more frequent in Caucasians, obese individuals, and those with a low income (1). The recurrence rate of urinary calculi is 50% within 10 years of the first episode (2). In addition to the conditions stated above, metabolic disorders such as hypercalciuria and hypocitraturia are typically involved in the genesis of urinary calculi (3).

Knowledge of the pathophysiological mechanisms involved and their risk factors, such as low urinary volume and high intake of calories, sodium and protein are important for modifying the natural history of the disease (2). Indeed, a reduction in the recurrence of urinary calculi of at least 50% can be achieved with dietary guidelines, lifestyle changes and the use of specific medications (4, 5).

In addition to conventional treatment for lithiasis, medicinal plants have long been used worldwide (6). *Phyllanthus niruri* or “stone breaker tea” is one such natural alternative that is inexpensive, easy to obtain and has a low incidence of adverse effects (7). To date, anti-inflammatory (8), anti-hyperuricemic (9), and diuretic properties have been described for this plant (10). Although many studies have shown the beneficial effects of *P. niruri* and its potential to inhibit the formation of kidney stones, clinical studies remain scarce (11, 12).

Thus, the aim of this study was to evaluate the effects of *P. niruri* on metabolic parameters in patients with urolithiasis.

This protocol was submitted to and approved by the Ethics Committee for Research Project Analysis of the Clinical Hospital, University of São Paulo, Medical School under number 0304/11. It was also approved and sponsored by the Foundation of Research Support of São Paulo (FAPESP) under number 12/50031-7.

## MATERIALS AND METHODS

The study was developed at the Urologic Division of the Clinical Hospital, University of São Paulo, Medical School. All patients included presented one or multiple stones smaller than 10mm. Diagnosis was based on ultrasonography or computed tomography (CT). The age of the patients ranged between 18 and 60 years.

Exclusion criteria included patients with a serum creatinine level >2.0mg/dL, urinary tract infection, non-controlled diabetes, chronic liver disease, cancer or pregnant women.

The study was divided into three stages: baseline, *P. niruri* and washout. The baseline stage was that prior to intervention. The *P. niruri* stage consisted of 12 weeks of ingestion of an infusion tea prepared with the dry extract of *P. niruri*, according to literature recommendations (13), a week of rest was included without using the plant after each week of consumption (2 weeks). The final step, called washout, involved 12 weeks without ingestion of *P. niruri*. The patients themselves were the controls, and each patient was followed for 26 weeks. The patients were subjected to clinical, anthropometric, serum and urinary metabolic analyses at all stages of the study.

Demographic data included age, race, sex, family history and medication use. Clinical data comprised systolic and diastolic blood pressure and anthropometric evaluation (weight, height, body mass index (BMJ). Serum biochemical and urinary analyses and renal ultrasonography were performed at baseline, immediately after *P. niruri* use and at the end of the washout period. Image evaluation was performed for all patients by the same radiology physicians who were blinded to the group composition.

Serum biochemical analysis was performed during the baseline, *P. niruri* and washout periods and included a complete blood count and assessment of urea, creatinine, sodium, potassium, glucose (fasting), uric acid, total and ionized calcium, beta human chorionic gonadotropin (HCG; females only), total cholesterol and fractions, triglycerides, alanine aminotransferase, aspartate aminotransferase, gamma-glutamyl transpeptidase, amylase and bilirubin levels. Serum samples were collected from all participants after fasting for 12 hours.

Twenty-four-hour urinary measurements included calcium, oxalate, citrate, uric acid, magnesium, sodium, potassium, creatinine, urea and phosphorus levels. Urinalysis with urinary pH measurement and a urine culture was performed using spontaneous voided urine.

The criteria for evaluation of metabolic abnormalities in 24-hour urine samples (mg/g creatinine) were hypercalciuria (calcium >240/>200mg/24-hour), hyperoxaluria (oxalate>40mg/24-hour), hypocitraturia (citrate<320/<290mg/24-hour), hypernatriuria (sodium >150mg/24-hour) hyperuricosuria (uric acid >0.75/>0.6g/24-hour), abnormal pH (<5.8 and >6.2) and low urine volume (<2L/day) for men and women, respectively.(4).

After receiving clinical, laboratory and abdominal ultrasound evaluations, the patients included in the study received a monthly visit and 60 sachets of *P. niruri* as a dry extract containing 4.5g of the herb. The patients were instructed to prepare an infusion containing 250mL of boiling water for each 4.5g herb sachet and to drink two sachets/day.

Extracts of *P. niruri* (family Euphorbiaceae) were obtained from qualified manufacturers and from medicinal plant laboratories, and we obtained physicochemical and microbiological analysis reports for all lots used in this study. The plant was produced in Brazil, subjected to a shade drying method, stored at a temperature of 15-35°C in a dry place and conserved with gamma irradiation to avoid contamination by fungi or bacteria. The minimum tannin content was 1.5%, and the moisture content was 17%. Indications for use in disorders of the urinary tract include renal lithiasis, cramps, cystitis and nephritis. The plant is known to have analgesic, anti-inflammatory and hepatoprotective properties.

The use of medications for hypertension or other diseases was not discontinued for the study or considered in the analysis. Only patients using potassium citrate were instructed to discontinue its use two months prior to initiation of the study to avoid interference with the results of 24-hour urine citrate dosing.

Statistical analysis was performed with SAS-Statistical Analysis Software Version 9. Continuous variables are presented as the mean and standard deviation, and categorical variables are presented as frequency percentages. We used analysis of variance (ANOVA) for repeated measures, which takes into account patients being evaluated at three different times (repeated measures) for comparing the results. Significant differences between periods or stages of evaluation were compared with Tukey's test and the McNemar test. The significance level was set at 5%.

## RESULTS

In total, 430 patients were enrolled and initially screened, and 75 patients were considered eligible according to the inclusion criteria. Fifty-six patients completed the study after a few dropped out due to work or personal problems, surgeries or other treatments for non-urological (breast carcinoma, biliary calculus) diseases.

Of the 56 patients who remained, 36 (64.3%) were women, and 52 (92.8%) were Caucasians. The mean age was 44±9.2 years (22-58 years), and the baseline BMI was 27.2±4.4kg/m^2^. Thirty patients (53.6%) had a family history of calculi, 53 (94.6%) were sedentary, 27 (48.2%) had hypertension, and 26 (46.4%) had metabolic syndrome.

Of the studied cohort, 26 patients (46.4%) were using antihypertensive drugs; one patient (1.8%) was under hypoglycaemic medication, and four patients (7.1%) were using anti-depressants, among other medications. Diastolic blood pressure showed a slight increase, though not significant, during the *P. niruri* period, reaching 76.0±10.5mmHg and then decreasing to 72.5±10.5mmHg (p=0.02) during the washout period.

Abdominal pain was reported by 37 (66.1%) patients during the *P. niruri* period; dysuria occurred in 11 (19.6%) cases, haematuria in eight (14.3%) cases, and nausea and epigastric pain in six cases each (10.7%). However, these symptoms did not lead to discontinuation of infusion use in any case.

No significant changes in serum measurements, except for a significant decrease in alkaline phosphatase after the use of *P. niruri* when compared to the baseline period (67.7±22.2x63.5±20.6mg/dL; p=0.017), were found.

The 24-hour urine analysis revealed only a significant increase in potassium levels between the basal and washout periods, from 47.3±16.7mg/24-hour to 56.2±21.8mg/24-hour (p=0.017). However, a trend towards increased urinary potassium was noted during the use of the tea.

Analysis of electrolytes adjusted for 24-hour urine creatinine (Cr) revealed an increase in potassium and magnesium levels. This increase reached statistical significance in the washout period, as summarized in Table-1.

**Table 1:** Urinary parameters in patients treated with *P. niruri*.

Measure	MEAN (SD)			ANOVA P	TUKEY		
	Baseline	mg/vol.24-hour *P. niruri*	Wash out		Baseline × *P. niruri*	Baseline × Wash out	*P. niruri* × Wash out
Calcium	202.7±116.1	211.8±96.2	190.3±92.6	0.401	0.849	0.675	0.371
Uric acid	0.5±0.2	0.5±0.2	0.5±0.2	0.875	0.870	0.990	0.934
Sodium	171.6±74.0	166.1±78.9	182.7±62.2	0.368	0.881	0.610	0.344
Urea	20.1±6.0	18.6±7.7	20.9±8.4	0.070	0.278	0.679	0.063
**Potassium**	**47.3±16.7**	**50.5±20.4**	**56.2±21.8**	**0.023**	0.536	**0.017**	0.192
Citrate	379.9±191.1	398.1±219.0	412.8±175.8	0.515	0.922	0.488	0.739
Oxalate	24.4±15.5	23.9±14.5	22.3±9.7	0.639	0.952	0.619	0.795
Creatinine	1.3±0.5	1.3±0.5	1.2±0.5	0.672	0.682	0.773	0.989
Phosphorus	755.3±308.8	792.4±324.6	684.9±281.1	0.090	0.817	0,265	0.085
Magnesium	73.8±35.4	85.6±37.6	82.3±37.6	0.114	0.113	0.299	0.864
*Mg/Cr	**58.0±22.5**	**66.1±23.4**	**69.1±28.6**	**0.012**	0.081	**0.012**	0.736
*K/Cr	**39.3±15.1**	**42.7±19.3**	5**1.3±34.7**	**0.009**	0.587	**0.008**	0.095
pH	6.1±0.9	6.0±0.9	6.1±0.9	0.6698	0.7916	0.9705	0.6663
Volume	1927±614.5	2028.8±790.6	2014.7±656.6	0.4361	0.4044	0.8567	0.7481

**SD** = standard deviation; **K** = potassium; **Mg** = magnesium; **Cr** = creatinine

In the baseline stage, hypernatriuria was the most frequent urinary disturbance, occurring in 34 patients (60.7%). Hypocitraturia and hypercalciuria were observed in 24 (42.8%) cases, hyperuricosuria in six (10.7%), hyperoxaluria in five (8.9%), and low urine volume in 31 (55.3%). Furthermore, the pH value changed in 21 (37.5%) patients.

Evaluation of patients with urinary abnormalities at the baseline stage showed a tendency towards an increase in urinary citrate among the hypocitraturic patients (211.8±123.7 to 322.3±145.8mg/24-hour, p=0.2193). In addition, oxalate was significantly reduced, from 59.0±11.7 to 28.8±16.0mg/24-hour (p=0.0002), among those with hyperoxaluria, and uric acid was significantly decreased, from 0.77±0.22 to 0.54±0.07mg/24-hour (p=0.0057), among hyperuricosuric patients (Table-2).

**Table 2:** Metabolic alterations in 24-hour period urine at baseline and when taking *P. niruri* and wash out.

Metabolic alteration	Measure Urine 24- hour	Reference	Baseline	MEAN (SD) *P. niruri* mg/ vol.24-hour	Wash out	ANOVA P
Hypercalciuria (n=24)	Calcium	<240/200*	300.3 ± 106.6	237.2 ± 70.7	227.5 ± 79,3	0.2619
**Hyperuricosuria (n=6)**	**Uric acid**	**<0.75/0.6** *	**0.77 ± 0.2**	**0.54 ± 0.1**	**0.56 ± 0.12**	**0.0057**
Hypocitraturia (n=24)	Citrate	>290/320*	211.8 ± 123.7	322.3 ± 147.3	345.3 ± 147.3	**0.2193**
**Hyperoxaluria (n=5)**	**Oxalate**	**<40**	**59.0 ± 11.7**	**28.8 ± 16.0**	**33.0 ± 4.4**	**0.0002**
Hypernatriuria (n=34)	Sodium	<150	211.6 ± 62.8	183.5 ± 92.0	200.6 ± 59.7	0.1770

* value reference for men/women;

SD =standard deviation

Ultrasonography evaluation performed in the three periods of the study revealed that the total number of stones decreased in 38 (67.8%) patients, as summarized in Table-3 (from 3.2±2.02 to 2.0±2.07) and size. In 10 patients (17.8%), no alterations in the number of calculi were noted, and in eight patients (14.3%), an increase in the number of upper urinary stones was observed after the *P. niruri* period. Some patients reported spontaneous stone passage between the 21st and 70th day of the *P. niruri* period: four patients eliminated six stones, and another five individuals reported the presence of sandy fragments in the urine during the *P. niruri* period.

**Table 3:** Number and size of upper urinary calculi in patients treated with *P. niruri*.

Measure	MEAN (SD)			ANOVA P	TUKEY		
	Baseline	*P. niruri*	Wash out		Baseline × *P. niruri*	Baseline × Wash out	*P. niruri* × Wash out
Total calculi (n)	3.2±2.0	2.0±2.1	2.2±2.2	**0.0005**	**0.0005**	**0.015**	0.672
Right kidney (n)	1.6±1.4	1.1±1.2	1.2±1.4	**0.0176**	**0.0176**	**0.201**	0.587
Left kidney (n)	1.6±1.4	0.9±1.1	1.0±1.1	**0.0003**	**0.0003**	**0.003**	0.938
Size (mm)	15.6±10.6	9.4±8.9	11.2±11.1	**0.0002**	**<0.0001**	**0.045**	0.206

**SD** = standard deviation; **N** = 56

## DISCUSSION

The formation of urinary calculi is associated with different risk factors. Its prevalence is high worldwide with an increase in morbidity and health costs in a number of countries. However, fully effective treatment and prevention have yet to be established, and the use of medicinal plants may be helpful as coadjuvants. The use of *P. niruri*, a very common plant found in different countries, has been shown to be a viable alternative, though more clinical studies are necessary.

In the present study, we sought to evaluate the use of *P. niruri* in patients with small urinary stones. When we analysed urinary variables, we found no significant changes in urinary volume for a 24-hour period with the use of an infusion of the plant. The urine volume was close to the minimum recommended in the literature, which is 2L/day (3, 4). The patients in this study exhibited average values of 1927mL, 2029mL, and 2015mL daily for the baseline, *P. niruri* and washout periods, respectively. It is possible that the patients were aware of the importance of fluid intake to prevent the formation of new calculi and were thus already consuming the quantity of liquid recommended at the beginning of the study; this would have resulted in no significant change in urine volume. The diuretic effects of *P. niruri* have been described in experimental studies (10, 15), though Nishiura et al. (11) reported different results in a clinical study.

The increase in the 24-hour urine electrolytes found in this study may be related to the decrease in the number of calculi during the imaging evaluation. Increases in urinary potassium and magnesium levels lead to alkalinisation of the urine and consequently to an increase in urinary citrate, a potent inhibitor of calcium stone formation (5).

Potassium can moderate the concentration of sodium in urine and promote the elevation of citrate, which acts to correct urinary pH and acidity, possibly contributing to an increase in calcium solubility (5). These changes may interfere with some stages of crystallization in urine, such as a reduction in the nucleation, growth and aggregation of calcium oxalate crystals (5, 14). We observed a significant increase in potassium in 24-hour period urine samples between the baseline and washout periods, which was also noted in the change in the potassium/g Cr 24-hour ratio and in the change in the magnesium/g Cr 24-hour ratio. The increased potassium and magnesium levels observed in the study patients may help to explain the normalization of metabolic changes observed following the use of *P. niruri*.

Urine pH in the sample urinalysis did not change throughout the study and remained at a mean value between 6.0 and 6.1.

Diastolic blood pressure showed a significant reduction between the *P. niruri* and washout periods (16). A report of a hypotensive effect of *P. niruri* can be found in the literature (8).

The calculus imaging evaluation performed showed a reduction in the number of calculi after the *P. niruri* stage, as shown in Table-3, considering that the number of stones shown on ultrasound was lower due to the fact the some patients probably passed some of them during the study.

Although the same method of evaluation was utilized in the study performed by Nishiura et al., those authors did not show a change in the number and size of calculi before and after the use of a *P. niruri* extract in individuals with urinary lithiasis (11). In contrast, it has been reported that *P. niruri* promoted a reduction in the number of calculi and their appearance in relation to the most fragile aspect of the calculi structure (7, 17-19).

Although ultrasonography is not considered a gold standard for the evaluation of small calculi, we performed this evaluation for the purpose of clinical monitoring of the patients because repeated CT scans in a short period of time can result in undue radioactivity exposure, which is not recommended (20).

Some patients experienced haematuria and abdominal pain. The exact cause of these events not was clear and continuous monitoring of patients at monthly visits was implemented as a precaution during the study. The haematuria and abdominal pain may be related to small calculi eliminated during that period, as patients observed the presence of sand fragments in the urine. Regardless, we cannot attribute the pain reported to the consumption of *P. niruri* because this symptom is very frequent in patients with lithiasis.

In this study, there were no changes in serum levels of liver enzymes, urea and serum creatinine. In previous experimental studies, no adverse acute or chronic toxic effects, such as kidney, heart, liver or neurological effects, were reported with the use of *P. niruri* (17, 18). In a human study, Wang et al. (21) found the normalization of liver enzymes when the plant was used in patients with chronic liver disease. However, Nishiura et al. (11) reported no change in serum and urinary parameters when analysing 69 patients and a control group with and without the use of *P. niruri* (11).

Most studies to date have focused on the hepatoprotective effects of *P. niruri*, and the genus Phyllanthus has been utilized in the treatment of various liver disorders (8). The reduction in alkaline phosphatase levels observed in this study was beneficial. In addition to showing that there was no toxicity with the use of the plant extract, this decrease may have contributed to the reduction in the number of calculi at the end of the study, because alkaline phosphatase is a biochemical marker of bone metabolism and may be involved in the mechanism of calculus formation in hypercalciuria (22).

According to our evaluation of patients with metabolic alterations at the baseline and *P. niruri* periods, a significant normalization of urinary uric acid and oxalate values occurred in those with hyperuricosuria and hyperoxaluria, respectively. Citrate levels tended to norma lize in the same cohort, though without statistical significance, likely due to the small sample size. Moreover, no significant change was noted for patients with hypercalciuria or hypernatriuria at the basal stage.

Overall, clinical studies with more patients are needed to validate the use of *P. niruri* in daily practice, particularly in patients with baseline urinary metabolic disorders. *P. niruri* is an abundant natural resource available in many countries, and it can reduce the health system expenses associated with conventional drugs, which are often inaccessible to the majority of the population for long-term treatment.

## CONCLUSIONS

*P. niruri* intake is safe and does not cause significant adverse effects or significant serum metabolic changes. The use of the tea of this plant increases urinary excretion of magnesium and potassium. Patients with specific urinary metabolic changes such as hyperuricosuria and hyperoxaluria may benefit from ingestion of this tea.
